# Fundamental limits incorporating logical reasoning into Shannon’s information theory

**DOI:** 10.1073/pnas.2525600123

**Published:** 2026-08-27

**Authors:** Luis A. Lastras, Jonathan Lenchner, Barry M. Trager, Wojciech Szpankowski, Mark S. Squillante, Chai Wah Wu, Ronald Fagin, Alexander Gray

**Affiliations:** ^a^IBM Research, Yorktown Heights, NY 10598; ^b^ Department of Computer Science, Purdue University, West Lafayette, IN 47907; ^c^ Faculty of Mathematics and Computer Science, Jagiellonian University, Łojasiewicza 6, 30-348, Kraków, Poland; ^d^Centaur AI Institute, Lincoln, CA 95648; ^e^IBM Research, San Jose, CA 95141

**Keywords:** information theory, mathematical logic, semantic communication, reasoning

## Abstract

A cornerstone of Shannon’s famous information theory is the idea of decoupling the meaning of a message from its efficient transmission. In this article, we propose an extension of Shannon’s communication model where the sender and receiver are assumed to have reasoning capabilities. In such a setting, we gain insights by coupling the fields of information theory and mathematical logic. Under the assumption that a message is coming from a stochastic source, Shannon’s theory establishes that the fundamental compression limit is the entropy of the source. Imagine, however, that we were not interested in the message itself, but rather, we were focused on the logical conclusions that one could derive from it. In this work, we obtain a closed-form expression for a fundamental compression limit in the presence of reasoning capabilities. Our expression is valid under various assumptions about what the sender and receiver know, providing initial answers to key questions such as how much the fundamental limit varies as one narrows or widens the amount of knowledge that is being transferred from sender to receiver, or how, surprisingly, such a fundamental limit remains the same even if the sender is unaware of what it is that a receiver already knows. We also offer practical algorithms that are empirically demonstrated to be significantly more efficient than alternatives that do not account for the existence of reasoning capabilities.

## Significance

The value of an information bit can range from consequential to unimportant, depending on what one can deduce from it: a bit from a reliable sensor that tells a car when to stop carries far more value than one from a faulty sensor producing random fluctuations. Yet today we design communication protocols without accounting for the potential advantages of reasoning over received bits. We present a theory, developed by connecting information theory and mathematical logic, that accounts for such reasoning. Compared to a classical information-theoretic treatment, this theory demonstrates substantial gains in communication efficiency across a diverse set of tasks where a receiver’s goal is to deduce all or a subset of the facts a sender possesses.

In his famous Lectures on Physics, Feynman imagined a cataclysmic situation in which all scientific knowledge is destroyed, and a sentence carrying the most information in the fewest words needs to be chosen for transmission to future generations of beings (1). His choice for that single sentence, that “all things are made of atoms—little particles that move around in perpetual motion, attracting each other when they are a little distance apart, but repelling upon being squeezed into one another”, arguably implies an enormous amount of knowledge for anyone willing to think carefully about it.

The essential point of this example is the power of reasoning and experimentation when seeded with the right critical information. Interestingly, Shannon’s information theory intentionally does not account for the presence of computational devices that can reason over received information. This was part of a more general and deliberate decision to ignore semantics, focusing purely on the statistical transmission of symbols rather than their meaning.

**Fig. 1.**
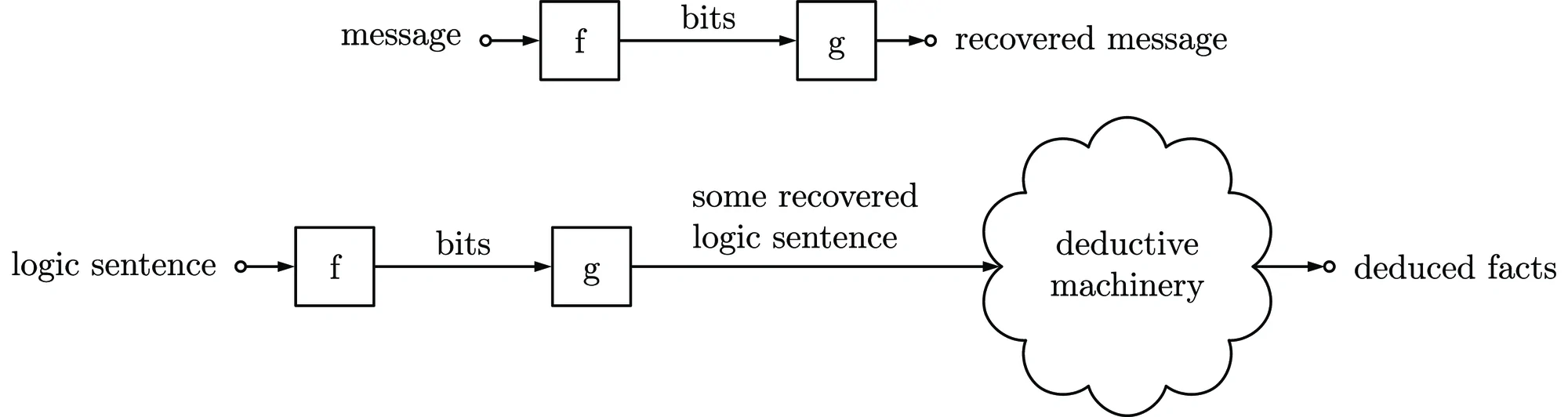
At the *Top*, Shannon’s original digital communication model. At the *Bottom*, a sketch of our proposed variant of Shannon’s model.

This paper proposes to extend that framework (Fig. 1) to account for reasoning by the receiver—specifically, logical deduction—which can dramatically amplify the effective content of transmitted information. We show that when senders and receivers can reason, significant efficiency gains over classical Shannon communication are achievable.

## Background.

In addition to its central role in communications, AI, and many other engineering fields, the formal framework of information theory has proved vital for modeling many of the most foundational scientific phenomena across the natural sciences.

It has been recognized within physics as a pivotal framework for understanding many core problems that cut across numerous areas of physics (2), including atoms in molecules (3), crystal structure (4), molecular interactions (5), and complex systems (6). It has been particularly essential in quantum computing (7), quantum physics more broadly (8), and in statistical thermodynamics (9, 10), to the extent that it was famously argued by Wheeler that all physical reality can be seen as a manifestation of the more fundamental notion of information (11). Particularly in the age of increasingly rich measurements and datasets, information theory has become an indispensable tool in biology for unlocking the fundamental principles underlying the biochemical signaling at the heart of the functioning of the cell (12–15), as well as advancing understanding in genomics (16–18) and drug response (19). Information-theoretic ideas also help to explain countless other scientific phenomena, from neuroscience (20–22) to human language (23) to art (24) to climate change (25). We expect the deep extensions to information theory described herein to provide correspondingly broad modeling insights across the sciences (26).

The success of notions of semantics-free information is associated with their wide generality. However, reintroduction of semantics has been of recurrent interest (27). In 1952, Carnap and Bar-Hillel (28) utilized the long-standing formalization of semantics as logic, which can be traced from Aristotle (logic as syllogism) through to Boole (Boolean logic) and Frege (First-Order Logic), to propose a mathematical conceptualization of a semantic information theory. In his 1953 paper on lattices and information theory (29), Shannon himself argued that entropy “can hardly be said to represent the actual information.”

In Carnap and Bar-Hillel’s work we find the key idea that if a sentence can be derived from the sentences one already knows, it conveys zero information. Similarly, in ref. 29, Shannon defined the “distance” between two sentences to be zero if each can be derived from the other. In both cases, the more general implications to communication systems were left unexplored. A number of proposals have since been put forward (30–43), broadly addressing one of three areas: the fundamental question of how to define semantic information, the transmission of semantic information, or the learning of models for it. In a subset of this work that has deeper connections to classical information theory (33–39), a common thread is the exploitation of Rate-Distortion theory (44), which Shannon introduced to extend the lossless compression results of ref. 45 to lossy coding (46). In these works, semantics are introduced by asserting that the fidelity criterion in lossy coding could be semantic in nature; they have not, however, explicitly considered reasoning in their frameworks. A different line of investigation (42, 43) starts from Shannon’s information lattice paper (29) and then constructs learning methods that are able to obtain explainable rules at different levels of abstraction. In another distinctive line of research, a theory of communication in the presence of goals has been put forth (40, 41).

AI has a long history of incorporating semantic understanding of the world into models, particularly through logic, ever since its origins in the 1950s (47). The rise and current dominance of semantics-free machine learning perhaps mirrors that of information theory, as technological developments have allowed it to achieve great successes but have also sparked discussion about the way ahead beyond current limits. This can be seen in an acceleration of interest in improving upon today’s deep learning models (which, in fact, heavily utilize classical information theory) toward those that encode semantics in various ways, as illustrated by Yann LeCun’s recent work (48) and the rapid growth of recent work in neuro-symbolic AI (49–51).

## Setup.

**Fig. 2.**
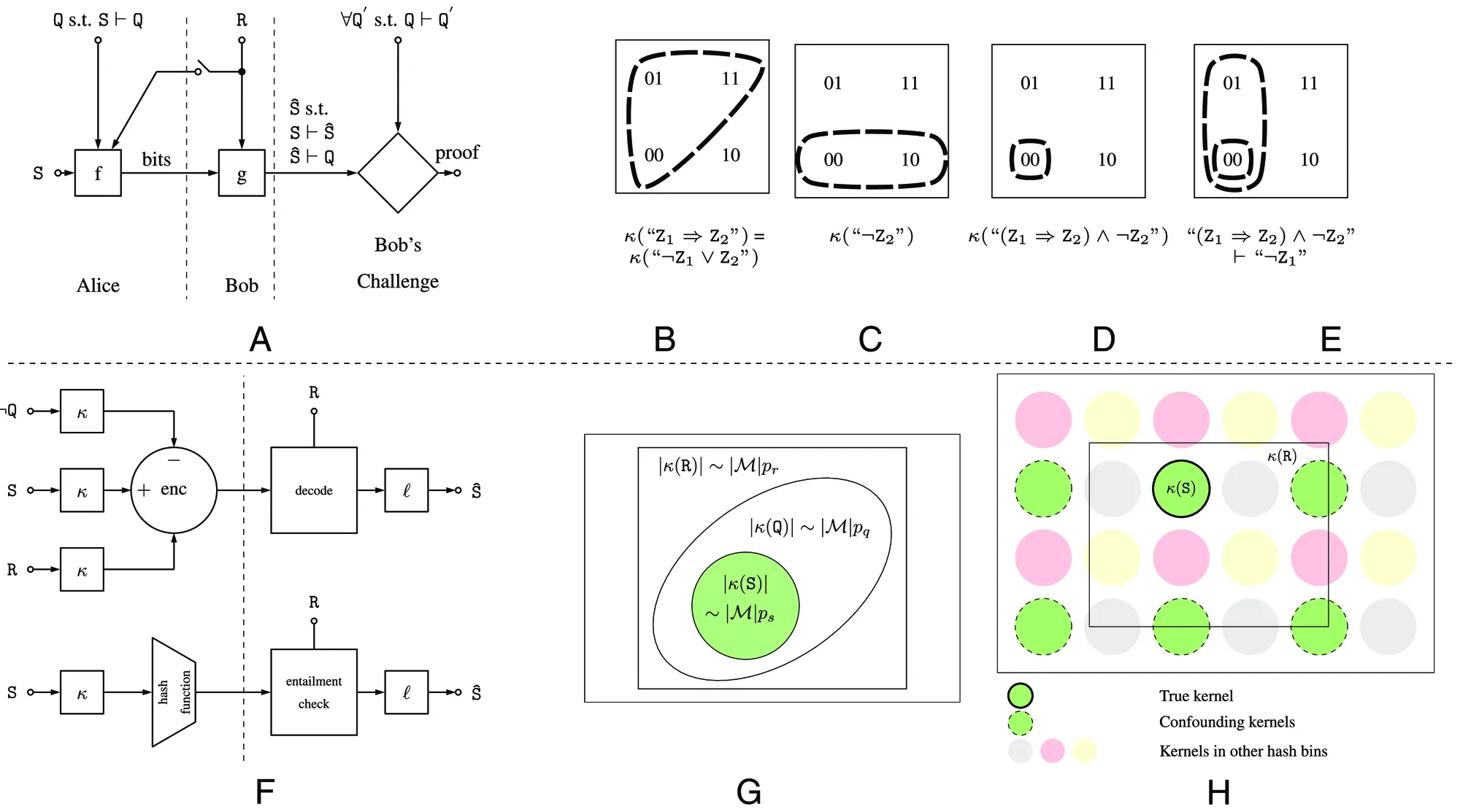
Communication problem setup and algorithm innovations. (*A*) General communication diagram which allows for Bob to possess facts that Alice may or may not know, and which allows for the query Q that Alice is helping Bob prove to be anything from her full knowledge S to a more targeted logic sentence. (*B*–*E*) Kernel diagrams respectively showing equivalence, negation, conjunction, and provable entailment for a two variable binary propositional logic setting $Z_{i}\in \left\{0,1\right\}$, i=1,2; here $Z_{i}=1$ (respectively, $Z_{i}=0$) denotes that the ith logic propositional variable is True (respectively, False). (*F*) Theoretically optimal solution architectures for the cases where Alice knows and does not know Bob’s knowledge, respectively. (*G*) Probabilistic model for kernels where the parameters $p_{s},p_{q},p_{r}$ are average kernel sizes, illustrated in the case Alice’s knowledge implies that of Bob. (*H*) No need to know decoding mechanism example where Alice mapped her kernel to a hash bin from 4 possible ones; Bob is able to reconstruct κ(S) without Alice knowing R ($p_{r}<1$) by rejecting all kernels that do not provably entail R and that do not match the received bin index.

The purpose of this section is to augment the general intuition given by Fig. 1 with a formal mathematical framework. We refer the reader to Fig. 2*A*, which shows a sender (Alice), a receiver (Bob), and sentences S, Q, and R that play different roles depending on the choice of setup. For now, the reader may assume that these sentences come from some, as yet unspecified, logical language L. The sentences S and R represent what Alice and Bob know to be true, respectively, and the query Q represents a fact that can be deduced starting from S; we say that Q is provably entailed by S, and write S⊢Q. The notion of provable entailment represents the cornerstone we use to bridge between the fields of information theory and mathematical logic. The choice of uppercase notation for these sentences underlines that we think of them as drawn from some distribution; the purpose of this is to be able to talk about expected performance across a class of problems.

Fig. 2*A* depicts the deductive machinery from Fig. 1 as a diamond. Consider a starting sentence:

A force of magnitude F imparts an acceleration on a mass of magnitude m equal to F/m (an expression more conventionally expressed as F=ma).

And a query to potentially be proved:

Given two objects dropped from above the earth’s surface at the same height, the time until impact is the same regardless of their mass, disregarding the effect of air friction.

The deductive machinery will either be able to prove the query from the starting sentence, or conclude that the query cannot be proved from the starting logic sentence. Importantly, however, the deductive machinery may have access to additional background facts, such as

The force imparted on either mass, $m_{1}$ or $m_{2}$, of a two-mass system with each mass at a distance r from one another is given by the equation $F=G\frac{m_{1}m_{2}}{r^{2}}$, where G is a constant.

The issues of i) what constitutes a valid sentence, given suitable syntactic rules, ii) how one defines what it means for a sentence to be true or false, and iii) how one deduces one sentence from another (or from another set of sentences) given a proof system, P, are at the foundations of mathematical logic. In an extended version of this article (52) we carefully construct a bridge between these foundational logical concepts and our theory of semantic communication. The provable entailment operator, ⊢, which we utilize informally here, is more formally defined with respect to a particular choice of a proof system, P, in ref. 52.

Now, imagine multiple objects with different masses at some distance from the earth. Each of these objects has a somewhat different force imparted on it based on its mass and the acceleration it experiences and additionally has its own time until impact. Each earth-object system is in this sense a “model” of the various physics sentences (the starting sentence, the query to be proved, and the background knowledge).

This notion of a model carries over to the formal logical theory we develop in ref. 52 where a model of a sentence is assumed to come from a set M of possible models. A logic sentence is thus intrinsically associated with those models within M that make the sentence true. The subset of models of a sentence S∈L is called the kernel of such a sentence and is denoted by κ(S). Notice that two different sentences from the language could be associated with exactly the same set of models, which makes those sentences logically equivalent.

This last observation is the key foundation for our contributions. Our work places very few restrictions on the choice of logic, language L and set M of models, with an important limitation that the latter currently needs to be finite. This includes propositional and first-order logic, but applies to additional logics and languages that we have yet to investigate. The limitation to a finite M was introduced to obtain the simplest possible nontrivial theory combining information theory and logic; we intend to remove such a limitation in subsequent treatments of this work.

For illustrative purposes, in Fig. 2 we consider propositional logic with 2 variables $Z_{1},Z_{2}\in \left\{0,1\right\}$, where $Z_{i}=0$ if the ith proposition is false and $Z_{i}=1$ if true. A set of models then coincides with a set of possible truth assignments to these variables. We depict three such kernels in Fig. 2*B*–*D*. Fig. 2*B* also illustrates how two different sentences that are logically equivalent have the same kernel, and Fig. 2*D* shows how the kernel of a conjunction of sentences is the intersection of the individual kernels. Fig. 2*E* illustrates a concept from mathematical logic that we use extensively in our article: A logic sentence provably entails another logic sentence if and only if the kernel of the former is a subset of the kernel of the latter. The concept of a kernel in the context of a semantic information theory traces its roots back to Carnap and Bar-Hillel, who defined a closely related concept using the word “range.”

### Initial meeting.

Alice and Bob meet ahead of time, and agree that the goal is for Bob to prove the truth of a logic sentence Q∈L using information that Alice will provide, employing a preagreed upon encoding. The sentences S,Q,R are not known at the time of this meeting, and will be revealed later to the relevant parties.

### Correlated world observations.

After this meeting, Alice and Bob go their own ways; Alice, the sender, obtains knowledge about the world summarized in a logic sentence S∈L whereas Bob, the receiver, obtains R∈L. We consider both settings where Alice knows and does not know R; we do assume that S⊢R except in an interesting incorrect information scenario discussed later. This provable entailment assumption roughly corresponds to a situation where Alice has made a superset of the observations about the world that Bob has made (but may not know exactly what subset Bob has), and where each has independently summarized their observations as logic sentences. The query Q is also revealed to Alice at this time, where we assume that S⊢Q; in words, Alice will help Bob prove something that she can prove with her knowledge. In the case Alice does not know R, we only present the case where Alice is equipping Bob to prove all she can (Q=S). In the case Alice does know R, we assume that Q⊢R. All three of S,Q,R are regarded as stochastic, with the general assumptions on their probability distribution to be described shortly; this is done so as to be able to talk about average performance.

### Communication.

In the case Alice knows R, she uses an encoder f(S,Q,R) to generate bits that are transmitted to Bob. In turn, Bob uses a decoder g(R,f(S,Q,R)) to obtain $\hat{S}$, which represents Bob’s updated logic sentence. In all cases, we assume that Bob ends up with knowledge consistent with that of Alice, written mathematically as $S⊢\hat{S}$ (but the reverse provable entailment may not always hold). In the more challenging setting where Alice does not know R (and we restrict Q=S), we allow for multiple communication rounds in which Alice and Bob exchange turns sending information, culminating again with Bob’s updated logic sentence $\hat{S}$. In either case, we use exactly the same figure of merit, defined more precisely below: the number of expected bits exchanged between Alice and Bob, and normalized by the number of models, where the expectation is with respect to S,Q,R for some distribution; once again, the general assumptions on this distribution will be described shortly. Note that the normalization by |M| is where we most overtly make use of the assumption of the finiteness of M; extensions to the infinite setting will obviously not rely on this specific type of normalization.

This setup has some similarities with Yao’s communication complexity (53), with one difference being that we do not identify ahead of time a function both Alice and Bob want to compute. The information-theoretic counterpart of Yao’s model, studied by Orlitsky and Roche (54), characterizes the minimum rate at which a sender must communicate so that a receiver can compute a function of their joint data; our setting differs in that the receiver’s goal is not to evaluate a prespecified function but to construct a proof of a logical query.

### Challenge and deduction.

After the communication takes place, Bob is challenged with any sentence $Q^{\prime }$ that can be proven by Q (including possibly Q itself), and Bob is able to produce a proof for that query using the logic sentences $\hat{S}$ and R; mathematically, for the system to have succeeded, it must be the case that $\hat{S}⊢Q$.

### Probabilistic model and figure of merit.

All of our results are phrased in terms of expectations over various stochastic quantities. The independent variables are normalized expectations of kernel sizes; specifically, $p_{s}=E\left[|\kappa (S)|\right]/\left|M\right|$, $p_{q}=E\left[|\kappa (Q)|\right]/\left|M\right|$, and $p_{r}=E\left[|\kappa (R)|\right]/\left|M\right|$, where $0<p_{s}\leq p_{q}<p_{r}\leq 1$. For illustration purposes, in the setting of propositional logic with two variables, the normalized sizes of the kernels in Fig. 2*B*–*D* are 3/4, 1/2, and 1/4, respectively.

The figure of merit for our results is the minimum number of expected bits exchanged, normalized by |M|, defined as

[1] $$\begin{array}{c} \min_{f,g}E\left[\mathrm{len}(f(S,Q,R))\right]/\left|M\right|, \end{array}$$

where len(·) returns the length of the binary string that is passed to it, and the minimization is over all possible encoder and decoder pairs f,g that satisfy the provable entailment condition g(R,f(S,Q,R))⊢Q in the case Alice knows R. When Alice does not know R, the setup is more complex; this is partially treated in An Algorithm for the Case That Alice Does Not Know What It Is That Bob Knows, with additional details in *SI Appendix*, and fully described in ref. 52.

We will present a theoretical result in the form of upper and lower bounds on Eq. **1** that agree with each other asymptotically as |M| goes to infinity; for this reason, we refer to our result as the fundamental limit. Our upper bounds are applicable to any possible distribution over S,Q,R as long as the provable entailment conditions described earlier are met; these are illustrated geometrically in Fig. 2*G*.

For our lower bounds, we use a stronger assumption that can be roughly described as one where the elements of a kernel are chosen independent and identically distributed (i.i.d.) with some probability, from the elements of a subsuming kernel, resulting in the same overall expected sizes as before; in this case, $p_{s},p_{q}$, and $p_{r}$ can be more readily interpreted as probabilities. The precise definitions can be found in Conditions for the Validity of the Lower Bound of Theorem 1. In the case Alice does not know Bob’s R, the model by assumption is simpler: Only S and R are relevant; we thus set Q=S and $p_{q}=p_{s}$.

## Overview of Results.

All of our results are phrased in terms of a type of scaled conditional entropy that we call the logical semantic entropy, denoted by Λ, which increases monotonically in each variable:

$$\begin{array}{c} \Lambda \left(a,b\right)=a\log_{2}\left(\frac{a+b}{a}\right)+b\log_{2}\left(\frac{a+b}{b}\right). \end{array}$$

The identification of the role of this function in problems of efficient communication assuming reasoning capabilities is a key part of our contribution. We note that the same functional form appears independently in recent work on constrained entropy maximization (55, 56), where Λ(a,b) governs the entropy cost of merging two probability atoms of sizes a and b, and bounds the approximation gap for the NP-hard problem of entropy-bounded information maximization. In both their setting and ours, Λ emerges as the fundamental cost of a binary separation within a weighted structure, suggesting a deeper role for this function in information theory.

We are now in position to state our main result, to be interpreted in the context of the above setup.

Theorem 1. For any distribution over (S,Q,R) meeting the provable entailment conditions S⊢Q and Q⊢R, if the corresponding kernels have expected normalized sizes $p_{s},p_{q},p_{r}$, respectively, then for the case Alice knows what R is, an algorithm exists with normalized expected cost in bits that is upper bounded by $\Lambda \left(p_{s},p_{r}-p_{q}\right)+{O(|M|}^{-1}\log|M|)$. Under additional constraints (*Methods*), the normalized expected cost of any algorithm is lower bounded by $\Lambda (p_{s},p_{r}-p_{q})$. In the case Alice does not know what R is, under the additional assumption that Q=S and thus $p_{q}=p_{s}$, the same conclusions hold, where the figure of merit accounts for all communication in both directions.

A sketch of the proof of this Theorem is given in *SI Appendix*, with the full proof provided in ref. 52. We reiterate that the class of algorithms considered for the lower bound is large, as the result shares similar proof techniques with its corresponding classical information-theoretic cousins. Of note as well is that the upper and lower bounds match up to the approximation dependent on the |M| parameter, and that the same expression Λ is being used for either of the two settings considered in this article. We also note to the reader that the result above already contains significant advantages with respect to communication protocols that do not rely on reasoning; this will be argued in the remainder of this article.

### Solution architecture.

In Fig. 2*F*, we illustrate two architectures that achieve the upper bound in this theorem for the cases where Alice knows and does not know R, respectively. In both cases, we first map the sender’s logic sentence S to its kernel κ(S). When Alice does know R and the query Q may be more targeted (still respecting the assumption κ(S)⊆κ(Q)), the key technique involves approximating the kernel of Q with another kernel that has two properties: a) it is included in κ(Q), and b) it includes κ(S); such an approximating kernel is chosen from a carefully crafted list of kernels and its index is transmitted to Bob. This procedure is illustrated as the circle labeled “enc.” When Alice does not know R, the key idea is compressing Alice’s kernel by sending only the index of a bin (hash bin) to which the kernel is mapped by hashing. Bob is able to recover the kernel by choosing the one from the hash bin that provably entails R, thus eliminating confounding ones (Fig. 2*H*). To ensure such hash collisions occur with low probability, while maintaining very good compression, the hash bins need to be sized “just right.” This is attained by multiple preliminary communication rounds in which sender and receiver communicate their kernel sizes. The small probability event where this protocol runs into difficulties is addressed with one optional final round in which the sender kernel is transmitted with a simple encoding. This small probability can, in effect, be made vanishingly small as |M| grows. The process ends by using a function ℓ that is able to translate a kernel back to an exemplary logic sentence $\hat{S}$. Arguing why and how these techniques result in asymptotically optimal systems is addressed in *Methods* for a specific choice of parameters, with a sketch of the proof provided in *SI Appendix* and proven more generally in ref. 52.

### Empirical validation.

**Fig. 3.**
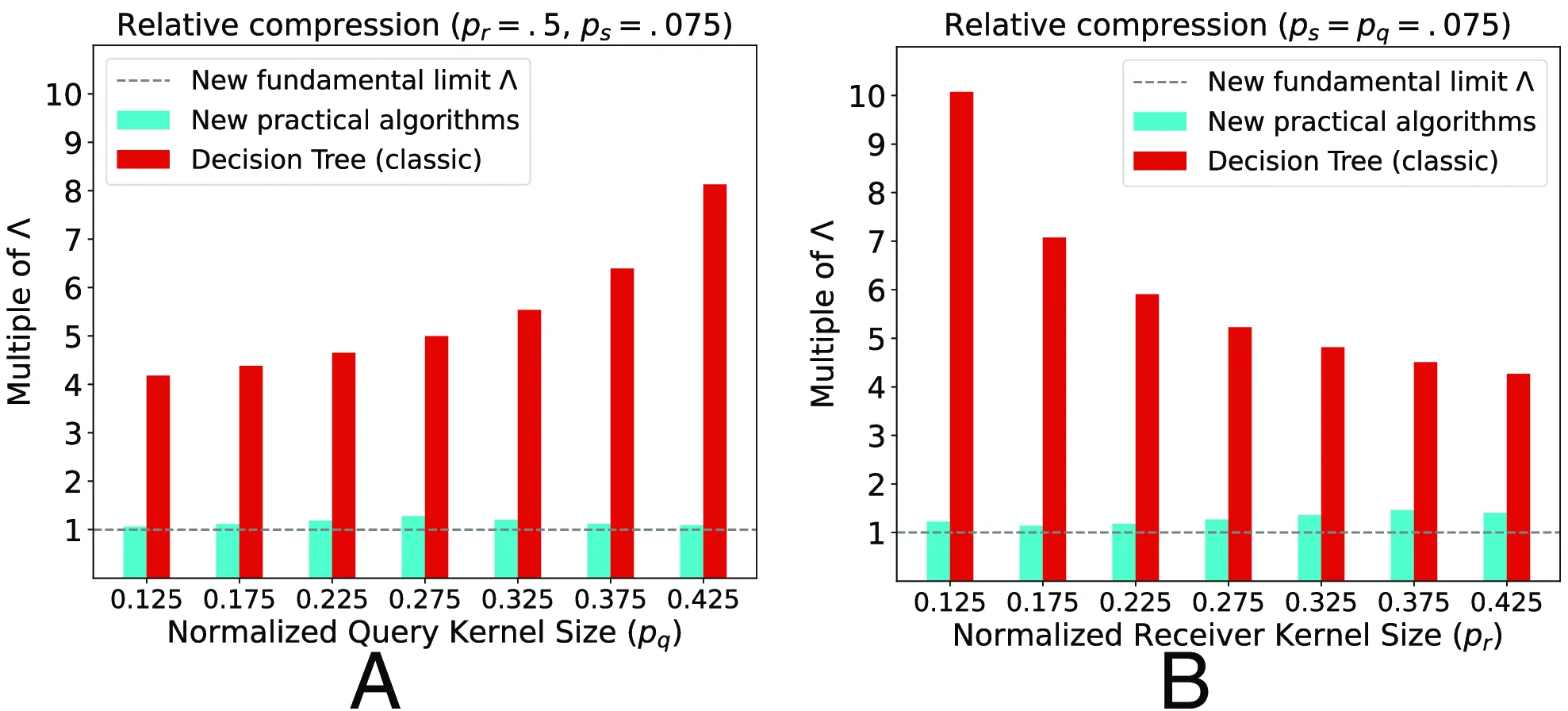
Experimental results for two scenarios. (*A*) Results as a multiple of the fundamental limit for the case Alice knows R, $p_{r}=0.5,p_{s}=0.075$ and $0.125\leq p_{q}\leq 0.425$, demonstrating the significant gains in communication cost using practical semantic communication algorithms compared to purely classical approaches. (*B*) Results as a multiple of the fundamental limit for the setting $p_{r}<0.5$ and $p_{s}=p_{q}=0.075$ in the case Alice does not know R, also comparing a classical approach with one leveraging logical semantics.

In Fig. 3, we present experimental results of practical algorithms for two contrasting situations. On the left, Alice and Bob have access to R, and Alice will help Bob prove a sentence Q, which is narrower than S. The sentence Q has an expected normalized kernel size of $p_{q}$, which we vary in the experiment. The red bars correspond to a coding technique based on an optimized representation (decision trees, explained in *SI Appendix*, *Method of Generating Generalized Decision Tree Sentences*) of either the sender or query logic sentences, whichever is cheapest after being passed through a good off-the-shelf lossless compressor (classical compression). The light-blue bars correspond to practical algorithms that we describe in Algorithms for Targeted Queries Based on Linear Algebra and An Algorithm for the Case That Alice Does Not Know What It Is That Bob Knows, and which leverage the mathematics of Galois fields (57). Both sets of bars are plotted relative to the ultimate information-theoretic bound Λ, shown by the dashed line.

Our practical algorithms use a combination of ideas based on linear algebra over the Galois Field with two elements {0,1} together with the exploitation of classical coding techniques as described in *Methods*. To implement the competing classical approaches, we developed an optimized representation of logic sentences based on decision trees (58–60).

Of note is the significant savings possible by using techniques that take advantage of logic sentences and reasoning. In the data compression field, significant advances are often measured in improvements of a few percentage points whereas here we are illustrating gains in integer multiples.

### No need to know.

A striking consequence of *Theorem* 1 is that, in this model, the same communication-cost limit applies regardless of whether or not Alice knows what it is that Bob knows. This phenomenon is also present in lossless classical communication (61), but it is rarer in the case of lossy compression (62, 63). In our work, communication is inherently lossy, in that the logic sentence $\hat{S}$ reproduced by Bob need not resemble Alice’s S nor Q.

### The less is more paradox.

**Fig. 4.**
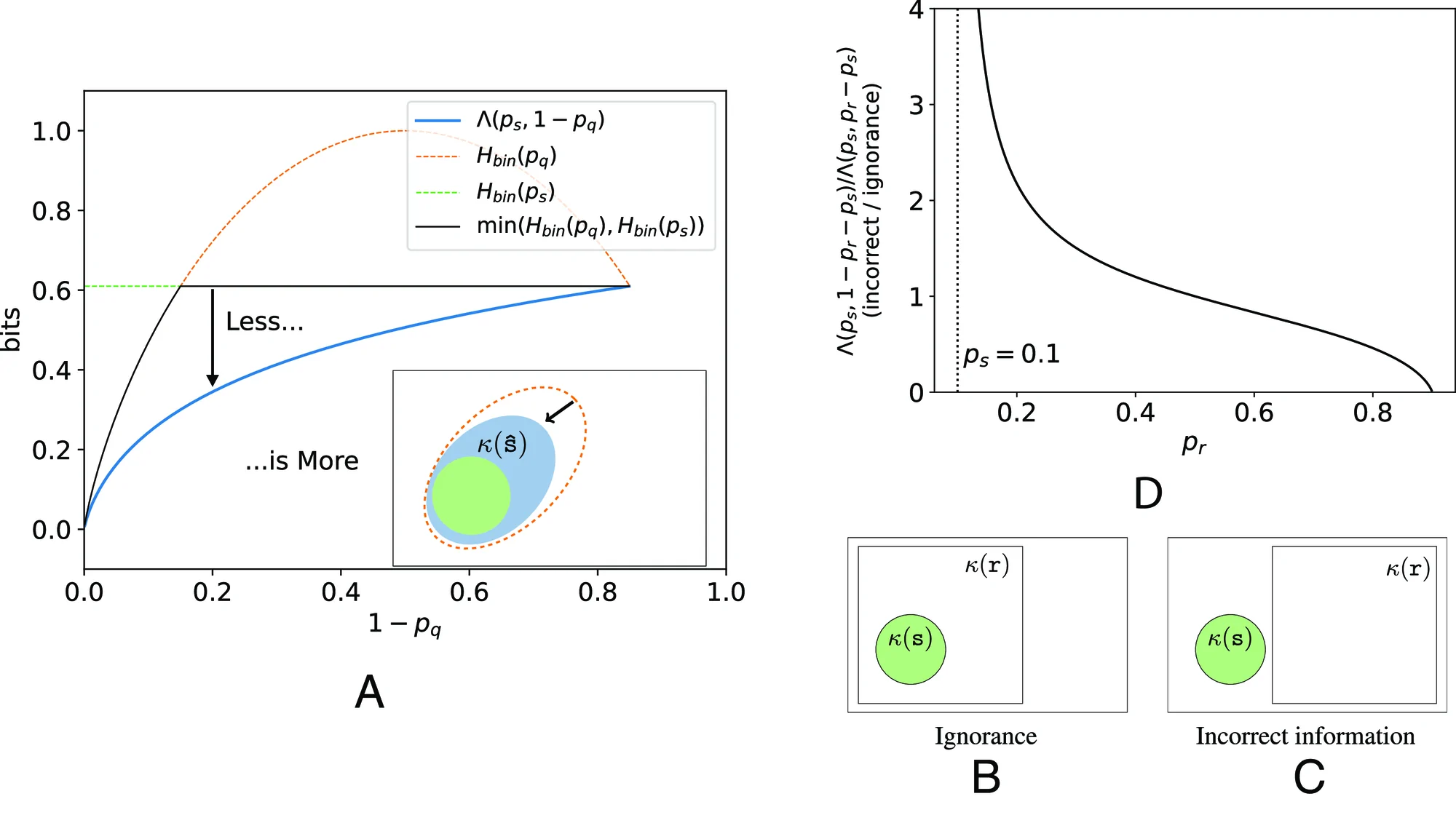
(*A*) Less is More. In blue, the ultimate communication limit Λ(·,·) for the case $p_{r}=1$, as the query ranges from trivial ($p_{q}=1$) to coinciding with the sender’s information ($p_{q}=p_{s}=0.15$). Λ(·,·) is cheaper (Less...) than the two obvious strategies, yet the kernel size received by Bob is smaller than that of the query, showing Bob can prove even more things (is More...) than required. A similar picture will hold for any $p_{r}$. (*B* and *C*) Kernel diagrams that model Bob’s state of ignorance versus incorrect information. (*D*) Relative cost of incorrect information to ignorance. We compare the ultimate limits for (*B*) (ignorance) and (*C*) (incorrect information); as Bob becomes more opinionated ($p_{r}\to p_{s}$, $p_{s}=0.1$), the ratio goes to infinity.

This is the scenario of a query Q that, while provably entailed by S, is not equivalent to S. Our result holds in the case Alice knows R, for every choice of $p_{s},p_{r}$, and $p_{q}$ such that $0<p_{s}<p_{q}<p_{r}\leq 1$. The fundamental bound in this case is $\Lambda (p_{s},p_{r}-p_{q})$, which is in general more efficient than sending either S or Q, which would respectively cost $p_{r}H_{\mathrm{bin}}\left(p_{s}/p_{r}\right)=\Lambda \left(p_{s},p_{r}-p_{s}\right)$ or $p_{r}H_{\mathrm{bin}}\left(p_{q}/p_{r}\right)=\Lambda \left(p_{q},p_{r}-p_{q}\right)$. In the *Upper* part of Fig. 4*A*, we plot the information-theoretic limit in this scenario (in blue) and compare it against the bits that would be required to send either the query or the sender information, for the case $p_{r}=1$. The fundamental limit is lower than both of these (Less...). Yet, when one examines the nature of the solution, one realizes that Bob obtains a sentence $\hat{S}$ whose kernel is generally smaller than that of Q, demonstrating that Bob is able to prove more than what he needed (...is More). The paradox is explained by noting that the way this is achieved is through deciding, ahead of time, on a terse collection of approximating kernels each of which can be used to solve the problem for multiple combinations of S and Q, further reducing the communication cost; a small concrete example showing this phenomenon is given in *SI Appendix*, *An Example of a Deterministic, Optimal Algorithm for a Small Problem with Targeted Queries*. Of note, the same paradox can be seen as having potential implications for security—being as efficient as one can to allow Bob to prove Q using facts consistent with Alice’s S results in revealing more than Q. In another play on words, one may say that one needs to say more to say less.

### The price of incorrect information.

In a line of work not directly covered by *Theorem* 1, but exploiting the same ideas, we introduce a rudimentary model of incorrect information. Formally, we modify the setup as follows. The entailment assumption S⊢R is replaced by an inconsistency assumption:

κ(S)∩κ(R)=∅,

meaning that no model simultaneously satisfies both Alice’s and Bob’s sentences. We assume Alice knows R, and that both parties agree Alice possesses the correct facts. The system succeeds if Bob obtains $\hat{S}$ logically equivalent to S, i.e., $\kappa (\hat{S})=\kappa (S)$. The figure of merit remains $\min_{f,g}E\left[\mathrm{len}(f(S,R))\right]/\left|M\right|$, with the probabilistic model unchanged except that the disjointness constraint requires $p_{s}+p_{r}\leq 1$ (Fig. 4*C*). In contrast, for the setting studied elsewhere in this article (Fig. 4*B*), Bob may be said to be in a state of ignorance, where κ(S)⊆κ(R), rather than having incorrect information.

Clearly this model is an unrealistic cartoon of the complex problem of incorrect information; we introduce it here merely to explore the type of conclusions one can derive from it, fully aware of its limitations; see ref. 32 for an early occurrence of this general type of problem. The limit $\Lambda (p_{s},1-p_{r}-p_{s})$ can be understood by observing that disjointness implies κ(S)⊆κ(¬R); that is, the negation of Bob’s incorrect sentence, with normalized kernel size $1-p_{r}$, serves as effective background knowledge logically consistent with Alice’s S. Applying the same reasoning as in *Theorem* 1 with this substitution yields the result. This is admittedly a crude bound—the negation of Bob’s beliefs is far from ideal as background knowledge—but it suffices to illustrate how the cost of correction escalates dramatically. For ignorance, as previously discussed, the limit is $\Lambda (p_{s},p_{r}-p_{s})$. We plot their ratio as a function of $p_{r}$ (Fig. 4*D*) showing that as Bob becomes more opinionated—meaning $p_{r}$ decreases toward $p_{s}$, so that his (incorrect) sentence imposes more restrictions on the world—the relative cost of correcting incorrect information versus ignorance grows to infinity, a result that appears to agree with common intuition.

## Methods

In this section we first describe the development of the practical algorithms used in our experiments. We then describe the experimental setup, and provide an expanded discussion on the experimental results. These experimental methods can attain the ultimate limits identified in this work only under very specific circumstances, leaving open the task of finding algorithms that approach such limits more generally.

### Overview of Algorithms.

We have devised two practical algorithms that are components of our proposed extended communication system, which are respectively aimed at the following scenarios:

- The “targeted query” scenario where both Alice and Bob know R and Alice must send the minimum amount of bits to Bob to allow him to prove a query Q that she can prove; and
- The scenario where Bob knows R but Alice does not know it; in this case the goal is for Bob to be able to prove all that Alice can while making the total exchange of bits between them, potentially over multiple turns and in any direction, as small as possible.

The algorithms for these two scenarios are based on the mathematics of linear algebra over the Galois Field (57) with two elements {0,1}, denoted by GF(2), and are shown to be optimal in specific settings. We then present experimental results comparing the performance of an ensemble of methods, including those developed herein, and contrast them with the Λ bound in *Theorem* 1 as well as optimized methods that treat the logic expressions as strings in the classical information-theoretic sense.

All of our algorithms target the efficient representation and decoding of kernels directly, and thus assume the prior existence of functions that map logic sentences to kernels and vice-versa. Note that while kernels are, strictly speaking, subsets of M, it is possible to represent them as binary vectors in ${\left\{0,1\right\}}^{\left|M\right|}$. Thus all of our algorithms will be phrased as a means to find encoders and decoders with various desirable properties for such binary vectors.

For two discrete random variables A,B with joint probability mass function P(A,B), we respectively define entropy and conditional entropy as

$$\begin{array}{c} H\left(A\right):=E\left[\log_{2}\frac{1}{P(A)}\right],H\left(A\;|\;B\right):=E\left[\log_{2}\frac{1}{P(A\;|\;B)}\right]. \end{array}$$

Furthermore, we define Shannon’s binary entropy by

$$\begin{array}{c} H_{\text{bin}}\left(p\right):=\left\{\begin{array}{cc} -p\log_{2}p-\left(1-p\right)\log_{2}\left(1-p\right), & p\in (0,1),\\ 0, & \text{otherwise.} \end{array}\right. \end{array}$$

Recall that M is a finite set of models, and that the kernel of a logic sentence, κ(·), is the set of models that make that sentence true. We assume an enumeration of such models:

$$\begin{array}{c} M=\{\mu_{1},\ldots ,\mu_{\left|M\right|}\}. \end{array}$$

### Conditions for the Validity of the Lower Bound of Theorem 1.

In the design of our experiments, we seek to demonstrate the extent to which our practical methods can achieve the lower bound of *Theorem* 1. Here, we rigorously complete the statement of this theorem by specifying the conditions needed for the validity of this lower bound, which will inform how we design the experiments. First, recall that the upper bound of *Theorem* 1 holds in significant generality, assuming only that

- S⊢Q, Q⊢R (and therefore S⊢R).

Define, temporarily, the random vector $\vec{\kappa }\left(S\right)$ to be an indicator vector for the kernel of S, governed by the rule $\vec{\kappa }{\left(S\right)}_{i}=1$ if $\mu_{i}\in \kappa (S)$ and 0 otherwise, and similarly define the same for Q and R. The lower bound augments the above assumptions on the upper bound with the additional assumptions:

- The random tuples $\left\{{\left(\vec{\kappa }\left(S\right),\vec{\kappa }\left(Q\right),\vec{\kappa }\left(R\right)\right)}_{i}\right\}$ are i.i.d.;
- R and κ(S) are conditionally independent given κ(R).

### Algorithms for Targeted Queries Based on Linear Algebra.

Our treatment of the problem of targeted queries allows for a sentence R to be optionally shared by both Alice and Bob. We present the algorithm in the setting where there is no such R for didactic reasons; extension to the case where such a sentence is available to both Alice and Bob is relatively straightforward. The algorithm starts by Alice computing the kernels κ(S)⊆M and κ(Q)⊆M; Alice accomplishes her goal if she is able to send to Bob a subset of M that includes κ(S) and is included in κ(Q). In this algorithm, we let $X_{i}=1$ if $\mu_{i}\in \kappa (S)$, $X_{i}=0$ if $\mu_{i}\in {\kappa (Q)}^{c}$ and $X_{i}=2$ otherwise; this then defines a vector $X^{\left|M\right|}\in {\left\{0,1,2\right\}}^{\left|M\right|}$.

Notice that if we were able to find a vector ${\hat{X}}^{\left|M\right|}\in {\left\{0,1\right\}}^{\left|M\right|}$ which agrees with $X^{\left|M\right|}$ on all the positions $\left\{i:X_{i}\in \left\{0,1\right\}\right\}$, then Alice would accomplish her goal, since such a vector can be seen as denoting the kernel of a sentence that Bob will be recovering from Alice’s transmission; note that such a vector would in effect be a partition of the sets $\left\{i:X_{i}=0\right\}$ and $\left\{i:X_{i}=1\right\}$. Furthermore, to accomplish her goal economically, there must be a way to transmit this vector efficiently to Bob, where efficiency is quantified as the expected number of bits transmitted.

We now present a general algorithm for finding partitions that can be represented efficiently by exploiting linear algebra over GF(2). This algorithm can be applied even when we do not have any statistical model of the underlying sets for which we are creating a partition. In the special case where the entries of $X^{\left|M\right|}$ are drawn i.i.d. from {0,1,2} using probability masses ($p_{0},p_{1},1-p_{0}-p_{1}$), we will show that this algorithm is asymptotically optimal if $p_{0}=p_{1}$, when competing against the class of all possible algorithms, whether based on linear concepts or not. Note that there is no special practical significance to this case; the existence of algorithms achieving the lower bound of *Theorem* 1 in complete generality is proven in ref. 52, with a proof sketch given in *SI Appendix*.

The practical algorithm assumes that Alice and Bob share a means of creating identical pseudorandom bits independently; this can be easily achieved by initially agreeing to a pseudorandom number generator and a seed. For ease of analysis, we assume that such pseudorandom bits are i.i.d. bits uniformly distributed over {0,1}.

Taking |M| of these random bits we can construct a 1×|M| row vector which we shall denote by $G_{1}$. Taking another |M| of them, we can append a second row to $G_{1}$, leading to a 2×|M| matrix that we denote by $G_{2}$. By repeating this procedure an arbitrary, but finite, number of times, we can assume that Alice and Bob share $G_{r}$ for any r. In practice, the number of rows r we will be using, although variable, will be slightly larger than $\left|M|(p_{0}+p_{1}\right)$. Define

$$\begin{array}{c} \Psi :=\{i:X_{i}\in \left\{0,1\right\}\}. \end{array}$$

For any given vector $x\in {\left\{0,1\right\}}^{\left|M\right|}$ and set of indices ξ⊆{0,1,…,|M|−1}, let ${\left[x\right]}_{\xi }$ denote the |ξ|-long vector obtained by extracting from x only the indices given by ξ. The algorithm starts by finding the smallest positive integer J such that the equation

[2] $$\begin{array}{c} {\left[M\cdot G_{J}\right]}_{\Psi }={\left[X^{\left|M\right|}\right]}_{\Psi }, \end{array}$$

can be solved for some 1×J vector M with entries in GF(2); here, the matrix multiplication is interpreted to be using GF(2) arithmetic. Intuitively, such a solution can be found because the dimension of the row space of $G_{j}$ increases as j grows with high probability; thus this dimension will eventually become |Ψ|, rendering the equation always solvable; this observation will be shortly used formally.

Once Alice finds such a J, she first sends it and then sends M. We could represent J in base 2 and transmit such bits; however, this presumes that both sender and receiver know, ahead of time, how many bits are needed to represent J. A more general solution can be found from the literature of *universal codes for integers*; one such example is a code due to Elias (64) which represents an arbitrary positive integer j with

[3] $$\begin{array}{c} \left\lfloor \log_{2}j\right\rfloor +2\left\lfloor \log_{2}\left(1+\left\lfloor \log_{2}\left(j\right)\right\rfloor \right)\right\rfloor +1\\ \leq \log_{2}j+2\log_{2}\left(1+\log_{2}j\right)+1 \end{array}$$

bits, where the upper bound is valid for j≥1. Denoting this code as ${\text{elias}}_{\delta }\left(j\right)$, and the number of bits that such a code assigns to j by $\mathrm{len}({\text{elias}}_{\delta }\left(j\right))$, the sender then sends the integer J to the receiver using

$$\begin{array}{c} \mathrm{len}({\text{elias}}_{\delta }\left(J\right)) \end{array}$$

bits, followed by the J bits in the message M. The receiver then decodes J and computes $M\cdot G_{J}$ to retrieve the partition.

We now analyze the expected performance of this algorithm. We first observe that

[4] $$\begin{array}{ccc} & E[J+\mathrm{len}({\text{elias}}_{\delta }\left(J\right))]-1\\ & \overset{\left(a\right)}{\leq }E[J+\log_{2}J\;+2\log_{2}\left(1+\log_{2}J\right)] & \\ & \overset{\left(b\right)}{=}E[E[J+\log_{2}J+2\log_{2}\left(1+\log_{2}J\right)|\;\Psi ]] & \\ & \overset{\left(c\right)}{\leq }E[E\left[J\;|\;\Psi \right]+\log_{2}E\left[J\;|\;\Psi \right]+2\log_{2}\left(1+\log_{2}E\left[J\;|\;\Psi \right]\right)], \end{array}$$

where (a) follows from Eq. **3**, (b) follows from the law of total expectation, and (c) follows from the concavity of the logarithm. We next upper bound $E\left[J\;|\;\Psi \right]$. To obtain an upper bound for J, we note that a sufficient condition to be able to solve Eq. **2** is that ${\left[G_{J}\right]}_{\Psi }$ has full rank, that is, the dimension of the space spanned by the rows of ${\left[G_{J}\right]}_{\Psi }$ is exactly |Ψ|.

Let $W_{i}$ be the smallest integer such that the row subspace spanned by ${\left[G_{W_{i}}\right]}_{\Psi }$ has dimension i. We can trivially write

$$\begin{array}{c} W_{\left|\Psi \right|}=W_{1}+\sum_{i=1}^{|\Psi |-1}W_{i+1}-W_{i}. \end{array}$$

The probability that a vector drawn uniformly from $GF{\left(2\right)}^{\left|\Psi \right|}$ is nonzero, and therefore spans a space of dimension 1, is given by $1-2^{-|\Psi |}$; hence,

$$\begin{array}{c} E\left[W_{1}|\;\Psi \right]=\frac{1}{1-2^{-|\Psi |}}. \end{array}$$

We now analyze the expected difference $E[W_{i+1}-W_{i}\;|\;\Psi ]$. Note that, by definition, ${\left[G_{W_{i}}\right]}_{\Psi }$ spans a subspace of dimension i, which must consist of exactly $2^{i}$ elements. If one chooses an element from $GF{\left(2\right)}^{\left|\Psi \right|}$ uniformly at random, the probability that it lies within the subspace spanned by ${\left[G_{W_{i}}\right]}_{\Psi }$ is $2^{i-|\Psi |}$. As a consequence, the probability that the subspace spanned by the rows of ${\left[G_{W_{i}}\right]}_{\Psi }$ with such a random vector having dimension i+1 is $1-2^{i-|\Psi |}$, and thus

$$\begin{array}{c} E\left[W_{i+1}-W_{i}\;|\;\Psi \right]=\frac{1}{1-2^{i-|\Psi |}}. \end{array}$$

Observing that $J\leq W_{\left|\Psi \right|}$, we have

$$\begin{array}{c} E\left[J\;|\;\Psi \right]\leq \sum_{i=0}^{|\Psi |-1}\frac{1}{1-2^{i-|\Psi |}}\leq \left|\Psi \right|+2. \end{array}$$

Continuing from the inequality in Eq. **4**, and normalizing by |M|, we obtain the following upper bound on the bit cost:

$$\begin{array}{c} E[|\Psi |+\log_{2}\left(|\Psi |+2\right)+2\log_{2}\left(1+\log_{2}\left(|\Psi |+2\right)\right)+3]/\left|M\right|. \end{array}$$

To understand the quality of this algorithm, we assume that the entries of $X^{\left|M\right|}$ are drawn i.i.d. from {0,1,2} using probability masses ($p_{0},p_{1},1-p_{0}-p_{1}$) and therefore $E\left[|\Psi |\right]=\left|M\right|\left(p_{0}+p_{1}\right)$, which results in the following upper bound on average performance:

[5] $$\begin{array}{cc} p_{0}+p_{1} & +\frac{\log_{2}\left(|M|(p_{0}+p_{1})+2\right)}{\left|M\right|}\\ & +2\frac{\log_{2}\left(1+\log_{2}\left(|M|(p_{0}+p_{1})+2\right)\right)}{\left|M\right|}+\frac{3}{\left|M\right|}. \end{array}$$

A special case of mathematical, rather than practical, interest arises when $p_{0}=p_{1}$. The achievable limit for this setting, as unveiled in our work, is given by

$$\begin{array}{c} \Lambda \left(p_{0},p_{1}\right)=\Lambda \left(p_{0},p_{0}\right)=2p_{0}, \end{array}$$

which is asymptotically, as |M|→∞, the same performance as in Eq. **5** and thus our algorithm is asymptotically optimal. If $p_{0}\neq p_{1}$, then $\Lambda \left(p_{0},p_{1}\right)<p_{0}+p_{1}$ and thus our algorithm is not asymptotically optimal. For illustration purposes, the reader will find an example of an optimal code for a small setting in *SI Appendix*. In the extended article (52), we present an asymptotically optimal general construction based on random coding arguments.

### An Algorithm for the Case That Alice Does Not Know What It Is That Bob Knows.

As a parallel to the previous subsection, we start by assuming that Alice computes the kernel κ(S)⊆M and that Bob computes the kernel κ(R)⊆M. Alice will accomplish her goal if she is able to transmit κ(S) efficiently, somehow leveraging the fact that she knows that Bob knows an R with the property that κ(S)⊆κ(R), but not knowing anything further about R or κ(R) at the outset of the communication.

In this algorithm, we define the vectors $X^{\left|M\right|},Y^{\left|M\right|}$ through the following: $X_{i}=1$ if $\mu_{i}\in \kappa (S)$ and $X_{i}=0$ otherwise; similarly, $Y_{i}=1$ if $\mu_{i}\in \kappa (R)$ and $Y_{i}=0$ otherwise; note that, by assumption, $\left\{i:X_{i}=1\right\}\subseteq \left\{i:Y_{i}=1\right\}$. We assume that Alice knows $X^{\left|M\right|}$ but not $Y^{\left|M\right|}$ (other than the condition above), and that Bob knows $Y^{\left|M\right|}$. The goal is to efficiently transmit $X^{\left|M\right|}$ to Bob.

Let Δ>0 be an integer that is a design parameter. As in the previous algorithm using linear methods, we assume that Alice and Bob share a means for generating the same pseudorandom bits. In our analysis we assume that Alice and Bob share a matrix G with dimension (|M|+Δ)×|M| and entries drawn uniformly and independently at random from GF(2). We use the notation $G_{r}$ to denote the first r rows of the matrix G.

Let $\Psi =\{i:Y_{i}=1\}$. The method starts with Bob transmitting to Alice the integer |Ψ|. At this point both Alice and Bob will keep only the first |Ψ|+Δ rows of G, denoted by $G_{|\Psi |+\Delta }$. Alice sends to Bob the bits resulting from the matrix-vector multiplication $G_{|\Psi |+\Delta }X^{\left|M\right|}$, where $X^{\left|M\right|}$ is interpreted as a column vector. Let ${\left[G_{|\Psi |+\Delta }\right]}_{\Psi }$ denote the matrix obtained by extracting from $G_{|\Psi |+\Delta }$ the columns implied by the indices Ψ; note that this matrix is computable by Bob but not by Alice. Bob then attempts to solve the equation

[6] $$\begin{array}{c} {\left[G_{|\Psi |+\Delta }\right]}_{\Psi }z=G_{|\Psi |+\Delta }X^{\left|M\right|} \end{array}$$

for a unique z. If such a unique z exists, Bob can retrieve $X^{\left|M\right|}$ by making use of the fact that the only entries of $X^{\left|M\right|}$ which could possibly be equal to 1 must have an index contained in Ψ and the fact that

$$\begin{array}{c} {\left[X^{\left|M\right|}\right]}_{\Psi }=z. \end{array}$$

Therefore, $X^{\left|M\right|}$ can be recovered from z by lifting the latter using the indices Ψ.

By construction, Eq. **6** has at least one solution. If the |Ψ| columns of ${\left[G_{|\Psi |+\Delta }\right]}_{\Psi }$ are linearly independent, then that solution must be unique. The probability that these columns are linearly independent is given by $\prod_{i=0}^{|\Psi |-1}\left(1-2^{i-|\Psi |-\Delta }\right)$ and can be lower bounded in the following manner:


$$\begin{array}{l} \prod_{i=0}^{\left|\text{ψ}\right|-1}\left(1-2^{i-\left|\text{ψ}\right|-\text{Δ}}\right)=\text{exp}\left(\sum_{i=0}^{\left|\text{ψ}\right|-1}\text{log}\left(1-2^{i-\left|\text{ψ}\right|-\text{Δ}}\right)\right)\\ \geq \text{exp}\left(\sum_{i=0}^{\left|\text{ψ}\right|-1}-\frac{2^{i-\left|\text{ψ}\right|-\text{Δ}}}{1-2^{i-\left|\text{ψ}\right|-\text{Δ}}}\right)=\text{exp}\left(\sum_{i=1}^{\left|\text{ψ}\right|}-\frac{2^{-i-\text{Δ}}}{1-2^{-i-\text{Δ}}}\right)\\ \geq \text{exp}\left(\sum_{i=1}^{\left|\text{ψ}\right|}-2^{-i-\text{Δ}+1}\right)=\text{exp}\left(-2^{-\text{Δ}+1}\sum_{i=1}^{\left|\text{ψ}\right|}2^{-i}\right)\\ \geq \text{exp}\left(-2^{-\text{Δ}+1}\right). \end{array}$$

Bob can signal to Alice success or failure in finding a unique z with a single bit, and in the case of failure, Alice can simply send $X^{\left|M\right|}$ verbatim by sending |M| bits.

The expected number of bits transmitted in either direction is then upper bounded as follows:

$$\begin{array}{cc} \text{cost} & =1+\left(\;\text{probability of failure}\right)\left|M\right|+E\left[|\Psi |+\Delta \right]\\ & +E\left[\mathrm{len}({\text{elias}}_{\delta }\left(|\Psi |)\right)\right]\\ & \leq 1+\left(1-\exp\left(-2^{-\Delta +1}\right)\right)\left|M\right|+E\left[|\Psi |\right]+\Delta \\ & +E\left[\mathrm{len}({\text{elias}}_{\delta }\left(|\Psi |)\right)\right]\\ & \leq \left(1-\exp\left(-2^{-\Delta +1}\right)\right)\left|M\right|+E\left[|\Psi |\right]+\Delta \\ & +\log_{2}E\left[|\Psi |\right]+2\log_{2}(1+\log_{2}E\left[|\Psi |\right])+2. \end{array}$$

We now make the choice $\Delta =\lceil \log_{2}|M|\rceil$; using the inequality 1+x≤ exp (x) with $x=-2^{-\Delta +1}$ yields

$$\begin{array}{c} 1-\exp\left(-2^{-\Delta +1}\right)\leq 2^{-\Delta +1}\leq \frac{2}{\left|M\right|}. \end{array}$$

Normalizing this cost upper bound by |M| and defining $q_{1}:=E\left[|\Psi |\right]/\left|M\right|$, the normalized upper bound may be simplified to

[7] $$\begin{array}{c} q_{1}+O\left(\frac{\log_{2}\;\left|M\right|}{\left|M\right|}\right), \end{array}$$

as |M|→∞. To understand the quality of the above bound, assume temporarily that $Y^{\left|M\right|}$ has entries drawn i.i.d. from {0,1} using probability masses $\left(1-q_{1},q_{1}\right)$, and that each $X_{i}$ is drawn using the conditional distribution

$$\begin{array}{cc} P(X_{i}=1|Y_{i}=1) & =p_{1}/q_{1},\\ P(X_{i}=0|Y_{i}=0) & =1. \end{array}$$

Under these assumptions for $X^{\left|M\right|},Y^{\left|M\right|}$, a lower bound is given by ${\left|M\right|}^{-1}H\left(X^{\left|M\right|}|Y^{\left|M\right|}\right)=q_{1}H_{\text{bin}}\left(p_{1}/q_{1}\right)$. Comparing this lower bound to Eq. **7** we see that, if $q_{1}=2p_{1}$ and |M| is sufficiently large, then the linear coding algorithm can arbitrarily approach the lower bound; this observation is made for mathematical completeness as there is no special practical importance to this setting. For other choices of $q_{1},p_{1}$, nonetheless the algorithm is not optimal. As before, the existence of asymptotically optimal algorithms for all choices of parameters is proved in the extended article (52), with a proof sketch given in *SI Appendix*.

### Experimental Setup.

The goal of this section is to illustrate the possible gains that one may expect from a practical semantic communication system compared to a classical one, and to also show the existing gap of such a semantic communication system with respect to the ultimate bound given by Λ.

A first problem is the fact that it is possible to portray classical communication systems as nearly arbitrarily inefficient when compared to semantic ones. The reason is due to the multiple different ways in which sentences can express the same underlying semantic content; classical compression systems must be faithful to the original sentence itself whereas semantic systems, as treated in this article, can take advantage of the intended meaning of the symbols within the sentence. To illustrate this problem, given H(κ(S)|S)=0, we note that

[8] $$\begin{array}{c} H(S)=H(S\;|\;\kappa (S))+H(\kappa (S))\geq H(\kappa (S)). \end{array}$$

The gap H(S|κ(S)) can be very large; consider, for example, propositional logic in three variables $Z_{1},Z_{2},Z_{3}$ and observe that

$$\begin{array}{c} \begin{array}{c} {\text{Z}}_{3}\to ({\text{Z}}_{1}\wedge {\text{Z}}_{2})\\ ({\text{Z}}_{1}\wedge {\text{Z}}_{2})\vee \neg {\text{Z}}_{3}\\ \neg (\neg ({\text{Z}}_{1}\wedge {\text{Z}}_{2})\wedge {\text{Z}}_{3})\\ \neg ((\neg {\text{Z}}_{1}\vee \neg {\text{Z}}_{2})\wedge {\text{Z}}_{3})\\ \neg ((\neg {\text{Z}}_{1}\wedge {\text{Z}}_{3})\vee (\neg {\text{Z}}_{2}\wedge {\text{Z}}_{3}))\\ \neg ((\neg {\text{Z}}_{1}\wedge {\text{Z}}_{3})\vee (\neg {\text{Z}}_{2}\wedge {\text{Z}}_{3}))\wedge ({\text{Z}}_{1}\vee \neg {\text{Z}}_{1}) \end{array} \end{array}$$

are all logically equivalent sentences, yet appear different when interpreted as simple strings. One can expect semantic communication systems to take advantage of the observation in Eq. **8** and achieve a performance that is roughly H(κ(S)), but one must exercise caution and not overstate the benefit; for example, recent estimates (65) assert that in an average English sentence, about half of the bits can be attributed to establishing meaning [so to H(κ(S))], and the other half to the specific choice of wording [so, roughly, to H(S|κ(S))]. Our paper’s theoretical results not only leverage the phenomenon in Eq. **8**, but go beyond and exploit more delicate findings on how targeted queries may admit unusually efficient representations, or how, in some occasions, communication is surprisingly just as efficient when Alice does not know Bob’s sentence R as when she does know R.

Our experimental evaluation methodology follows the philosophy of always choosing a stronger “classical” (nonsemantic) baseline to compare against whenever a choice is on the table, even if these baselines start to become more semantic in nature. In particular:

- On purpose, we forego the type of advantage that stems from the observation in Eq. **8** even though we can legitimately claim it.
- Logic sentences will be optimized so that they can be represented more compactly by “classical” compressions systems.

#### Scenarios.

We demonstrate experimental results for the following two contrasting scenarios:

- **Targeted query with a shared sentence.** After the communication, Bob will be able to prove Q, which satisfies S⊢Q. Both Alice and Bob know R; only Alice knows S.
- **Alice does not know what it is that Bob knows.** After the communication, Bob will be able to prove all that Alice can. Only Bob knows R, and only Alice knows S.

These experimental results can be reproduced using the code and data in ref. 77

#### Test case generation.

For the purposes of our experiments, we first choose the underlying kernels of S,Q,R, and then generate the corresponding sentences; we stress that this is done for convenience and in no way reflects limitations on *Theorem* 1 which is stated for general distributions, particularly for the upper bound. We generated random kernels meeting all three constraints above on sets of 10 variables to test our methods in practice. For each of the kernels, we produced a generalized decision tree with the given kernel as its set of satisfying truth-value assignments. We then postprocessed each sentence by writing it using postfix notation, compressing variable names, and removing spaces. For the targeted query scenario, we have chosen $p_{r}=0.5$, $p_{s}=0.075$, and $p_{q}$ by sampling the range $\left[p_{s},p_{r}\right)$ uniformly. For the scenario where Alice does not know what it is that Bob knows, we set $p_{s}=p_{q}=0.075$ and let $p_{r}$ range in $\left(p_{s},0.5\right]$. We generated 1,000 test cases for each choice of $\left(p_{s},p_{q},p_{r}\right)$ considered. For specifics, we refer the reader to *SI Appendix*, *Additional Details on Experimental Methodology*.

## Experimental Results

### Semantic Communication Systems.

**Fig. 5.**
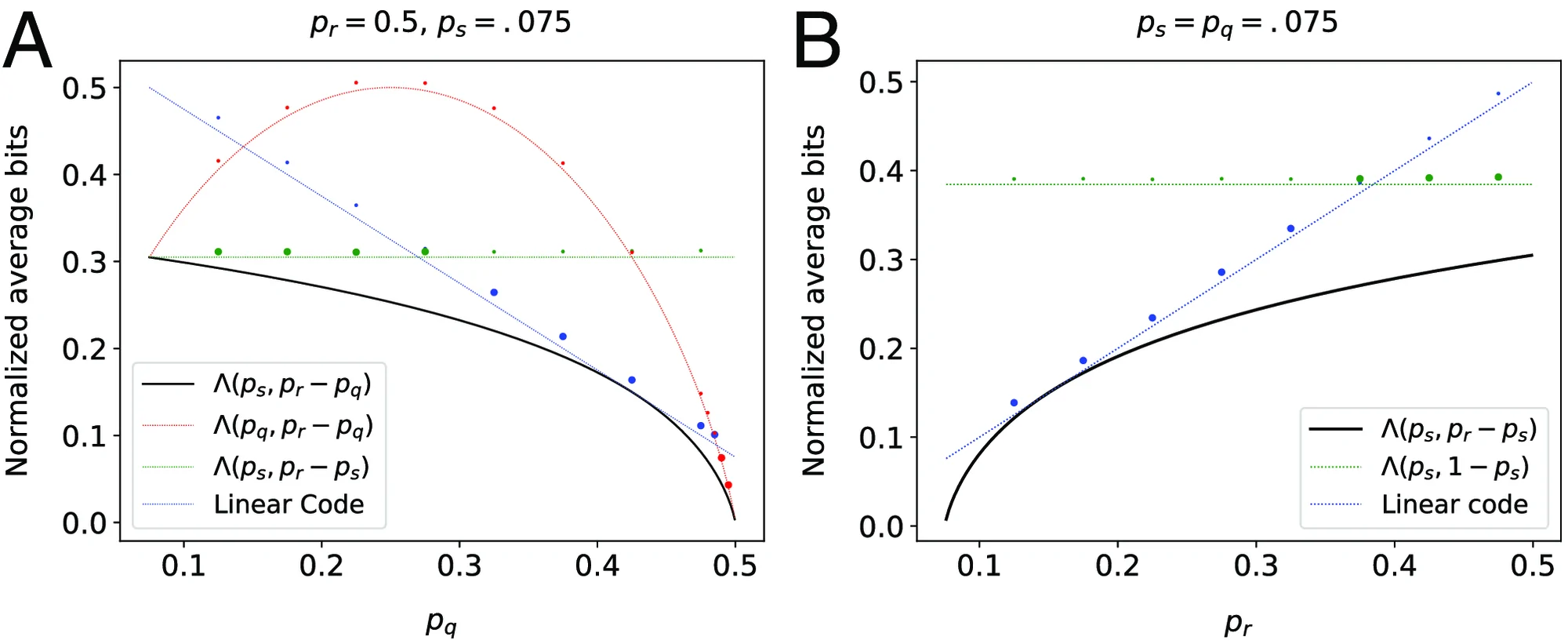
Comparison of practical semantic communication methods against the $\Lambda (p_{s},p_{r}-p_{q})$ limit derived in this work for two scenarios. (*A*) Semantic communication systems for targeted queries with a shared sentence with $p_{r}=0.5$, $0.075<p_{q}<0.5$. (*B*) Semantic communication systems for when Alice does not know what it is that Bob knows with $p_{s}=p_{q}=0.075$.

In a first set of experiments, we aimed to demonstrate the performance of our practical semantic communication techniques against the theoretical bounds. We have implemented the linear codes introduced earlier in this *Methods* section, which are used to address the two respective scenarios. Due to the fact that these linear codes are not always optimal, we augmented these systems with “näive” semantic communication strategies based on efficient lossless transmission of kernels. For certain parameter ranges these are better than the linear codes, and thus in such cases we can use them instead. In the case of targeted queries, the task of allowing Bob to prove Q can alternatively be accomplished by sending κ(Q) or κ(S) to him, whichever is cheapest. The results, averaged across all 1,000 test cases for each choice of parameters, can be found in Fig. 5*A* where in blue we illustrate the performance of linear codes, and in green and red we respectively illustrate the performance of sending κ(S) and κ(Q) losslessly using such efficient techniques. These methods rely on the idea of enumerating all possible kernels that can be transmitted and then computing a code directly from the integer representing the position in which such a kernel appears in the list, rather than the kernel itself. For an overview of these codes based on enumerative techniques, we refer the reader to ref. 66.

In each of these three practical systems, we include a curve of the same color that is a lower bound on performance for the specific technique. The gap between the dots and the lower bound curves is primarily due to the added cost of the Elias code which becomes negligible as the length of the code increases. The bolder dots are those that are closest to the fundamental limit derived in our article, which is the bold, lowest curve shown in the figure. Similarly, in the case that Alice does not know what it is that Bob knows, the task of allowing Bob to prove S can alternatively be accomplished by sending κ(S) to him, also using enumerative source coding (66). The results of this experiment can be found in Fig. 5*B*. Our practical codes can be quite efficient in certain scenarios, but additional work is needed to develop practical codes that come closer to the fundamental limits for wider ranges of $p_{q}$ and $p_{r}$; refer to Fig. 5 *A* and *B*, respectively.

### Classical Communication Systems.

In a second set of experiments, we aimed to contrast practical classical systems with semantic ones. In these experiments, we reused the best results from the practical methods above and compared them with classical compression systems.

We define classical compression systems as ones that may only use the sentence as presented to the communication system, and not perform operations on it with awareness of its underlying semantic content. These are examples of such systems:

- **Targeted query with a shared sentence.** Using the Decision Tree representation of a sentence, employ a standard compression algorithm (in particular, gzip, lzma, bzip2) to compress S and Q; choose the best representation and send that one.
- **Alice does not know what it is that Bob knows.** The same as above, but considering only S as in this scenario, Q=S.

We note that the classical system is forced to solve a fundamentally stricter problem, lossless reconstruction of the full sentence, because it lacks the capacity to make logical deductions. A system that has this capability can tolerate a lossy reconstruction as long as it is possible to deduce Q from R together with the information transmitted. We provided an additional advantage to the type of classical communication systems used above: We compressed all 1,000 samples simultaneously, which allowed the compression algorithms to leverage patterns that only emerge when more data are available. In contrast, the semantic communication systems compress just one instance at a time. We reiterate that by using Decision Trees, we have already used semantic concepts to benefit the classical baseline. The results corresponding to these experiments can be found in Fig. 3*A* and *B* above, as described in the main body of this article. These plots normalize the performance of the practical semantic or classical systems against the corresponding fundamental limit $\Lambda (p_{s},p_{r}-p_{q})$ and $\Lambda (p_{s},p_{r}-p_{s})$, respectively. We acknowledge that in the realm of classical communication systems we should consider Slepian–Wolf compression algorithms (61) as a baseline. This is particularly difficult to do as we are not aware of practical instances of such algorithms that would be applicable to the complex statistical distributions present in S and R; we leave this comparison for future research.

### Implications and Next Steps.

We have provided evidence that new communication system designs incorporating deductive inference mechanisms can be effectively analyzed and that the potential efficiency gains can be significant. To capitalize on this potential, nonetheless, additional progress is required.

First, our deductive inference-centered proposal can, in principle, be combined with other proposals (33, 34, 36, 37) that similarly rely on Shannon-theoretic principles, potentially unveiling even more communication efficiencies. Second, the communication model we introduced can be enhanced in many directions, including situations where Alice and Bob’s roles are more symmetric (they each know something useful to a joint goal (67)), adversarial (one may be trying to convince the other about something that is not true), consultant (Bob is seeking help for a specific matter), multiparty (one instructor, many students), and machine-oriented (Alice and Bob are AI agents). Sharper results that forego the expected cost assumption and instead hold for individual logic sentences are possible; we anticipate that techniques such as the ones developed in ref. 68 will be relevant in establishing these results.

We close with more speculative directions, each pointing to ways in which the interplay between logic and information theory may deepen. Just as standard information theory has provided mathematical foundations for machine learning (69), semantic information theory may provide analogous foundations for AI systems that incorporate logical reasoning. In ref. 52, we show that our results hold for Propositional Logic and First-Order Logic over finite models. We anticipate that our results will be extended beyond finite model theory, and to richer logics beyond First-Order Logic, for example to First-Order Logic with counting (70), Second-Order Logic (71, 72), or to logic capturing uncertainty (73–75). Given the close connections between Kolmogorov complexity and Shannon’s entropy (76), we anticipate connections between semantic information theory and computer programs. Each of these directions reinforces the broader thesis that reasoning and information are deeply intertwined, and that formalizing their interaction can yield both theoretical and practical dividends.

## Supplementary Material

Appendix 01 (PDF)

## Data Availability

All study data and software are included in ref. 77.
