# Supplementary material for: Fundamental limits incorporating logical reasoning into Shannon’s information theory
Source: Proc Natl Acad Sci U S A. 2026 Aug 27;123(35):e2525600123. doi: 10.1073/pnas.2525600123 (PMC13535204; doi:10.1073/pnas.2525600123)
Supplement: Supplementary file 1 [file pnas.2525600123.sapp.pdf]

Supporting Information for

Fundamental limits incorporating logical reasoning into Shannon's information theory

Luis A. Lastras, Jonathan Lenchner, Barry M. Trager, Wojciech Szpankowski, Mark S. Squillante, Chai Wah Wu, Ronald Fagin, Alexander Gray

Luis A. Lastras.
E-mail: lastrasl@us.ibm.com
Ron Fagin
E-mail: fagin@us.ibm.com

This PDF file includes:

Supporting text
SI References

Supporting Information Text

The main contribution of our paper is showing that the fundamental cost of communicating logical assertions—when both sender and receiver possess reasoning capabilities—is governed by the *logical semantic entropy* $\Lambda(p_s, p_r - p_q)$, a function that depends only on the normalized expected sizes of the kernels involved. This supporting information (SI) appendix provides a proof sketch for our main result (Theorem 1); the complete formal proof appears in (1). We also include a discussion about the significance of our results and a further explanation of our experimental methodology.

We recall the communication scenario considered. Alice possesses knowledge summarized in a logic sentence $\texttt{S}$ and wishes to help Bob—who possesses his own background knowledge $\texttt{R}$—prove a query $\texttt{Q}$. The provable entailment assumptions $\texttt{S} \vdash \texttt{Q}$ and $\texttt{Q} \vdash \texttt{R}$ ensure that Alice's knowledge is sufficient to prove the query, and that the query is a refinement of what Bob already knows—so Bob's background provides a relevant head start toward the proof goal.

The theorem addresses two settings:

1. *Alice knows Bob's background* $\texttt{R}$: Alice sends a single message to Bob; the cost is one-directional.

2. *Alice does* not *know* $\texttt{R}$ (with $\texttt{Q} = \texttt{S}$): Alice and Bob must interact—exchanging messages in both directions—to accomplish the goal; the cost accounts for all communication.

In both cases, the asymptotic normalized cost is $\Lambda(p_s, p_r - p_q)$, where p_s, p_q, p_r are the normalized expected kernel sizes of $\texttt{S}, \texttt{Q}, \texttt{R}$, respectively.

Notation and key tools. For a sentence $\texttt{s}$, its kernel $\kappa(\texttt{s}) \subseteq \mathcal{M}$ is the set of models satisfying $\texttt{s}$. Provable entailment $\texttt{s} \vdash \texttt{q}$ is equivalent to set inclusion $\kappa(\texttt{s}) \subseteq \kappa(\texttt{q})$. The logical semantic entropy is given by

$$\Lambda(a, b) = a \log_2 \left(\frac{a+b}{a}\right) + b \log_2 \left(\frac{a+b}{b}\right) = (a+b)\, H_{\mathrm{bin}} \left(\frac{a}{a+b}\right),$$

with the last equality connecting it to binary entropy $H_{\mathrm{bin}}(\cdot)$. A useful scaling property is $c \cdot \Lambda(a/c, b/c) = \Lambda(a, b)$.

The proof relies on two classical tools: (i) *Kraft's inequality*, connecting prefix-free code lengths to entropy; and (ii) *rate-distortion theory*, which provides both lower bounds (via mutual information minimization under distortion constraints—the "converse") and upper bounds (via random coding arguments that demonstrate achievability).

1. Case 1: Alice knows $\texttt{R}$

When Alice knows Bob's background, shared knowledge of $\kappa(\texttt{R})$ means that both parties can restrict their attention to models within $\kappa(\texttt{R})$. Alice's task is to specify $\kappa(\texttt{S})$—or, more generally, any intermediate set separating $\kappa(\texttt{S})$ from $\kappa(\texttt{Q})^c$—using as few bits as possible.

1.1. Lower bound: $\Lambda(p_s, p_r - p_q)$. The lower bound proceeds in two stages: first establishing the result for the special case $p_q = p_s$ (where Alice simply transmits her full kernel), and then extending to general p_q.

1.1.1. The case $p_q = p_s$ (no targeted query). We argue this by first asserting that any correct protocol must transmit at least $H(\kappa(\texttt{S}) \,|\, \kappa(\texttt{R}))$ bits and then showing that this conditional entropy equals $|\mathcal{M}|\, \Lambda(p_s, p_r - p_s)$.

1. *Every correct protocol pays at least* $H(\kappa(\texttt{S}) \,|\, \kappa(\texttt{R}))$. Three steps provide the bound.

   (Kraft's inequality). The expected length of any prefix-free code is at least the entropy of the codeword given $\texttt{R}$ (the sentence both Bob and Alice have access to):

   $$E[\mathbf{len}(f(\texttt{S}, \texttt{R}))] \;\geq\; H(f(\texttt{S}, \texttt{R}) \mid \texttt{R}).$$

   (Correct protocol). Using the transmitted bits $f(\texttt{S}, \texttt{R})$ and his own background $\texttt{R}$, Bob recovers a statement $\hat{\texttt{S}} = g(f(\texttt{S}, \texttt{R}))$ from which he can prove anything that Alice can prove. From this, it can be shown that $\kappa(\hat{\texttt{S}}) = \kappa(\texttt{S})$; by repeated applications of the fact that deterministic functions of random variables cannot increase entropy, we have

   $$H(f(\texttt{S}, \texttt{R}) \mid \texttt{R}) \;\geq\; H(\kappa(\texttt{S}) \mid \texttt{R}).$$

   (Markov chain assumption). Finally, we invoke the technical assumption that $\kappa(\texttt{S})$ and $\texttt{R}$ are independent given $\kappa(\texttt{R})$. In words, this says that any two receiver sentences sharing the same kernel are interchangeable from Alice's perspective: the specific syntactic form of $\texttt{R}$ beyond its semantic content carries no information about $\kappa(\texttt{S})$. This conditional independence assumption together with an inequality known as the "data processing inequality" in information theory are then used to obtain:

   $$H(\kappa(\texttt{S}) \mid \texttt{R}) = H(\kappa(\texttt{S}) \mid \kappa(\texttt{R})).$$

2. *Computing* $H(\kappa(\texttt{S}) \,|\, \kappa(\texttt{R}))$. The entailment $\texttt{S} \vdash \texttt{R}$ means that models outside $\kappa(\texttt{R})$ are automatically absent from $\kappa(\texttt{S})$. Now choose a set of models $k \subseteq \mathcal{M}$. For each model $\mu \in k$, the independent and identically distributed (i.i.d.) assumption provides (see *Conditions for the validity of the lower bound of Theorem 1* in the experimental section of the main paper):

$$P(\mu \in \kappa(\texttt{S}) \mid \kappa(\texttt{R}) = k) = \frac{p_s}{p_r}$$

independently as one varies μ. Each such Bernoulli(p_s/p_r) bit contributes $H_{\text{bin}}(p_s/p_r)$ to the conditional entropy. Summing over the $|\mathcal{M}|\, p_r$ positions in $\kappa(\texttt{R})$ on average yields

$$H(\kappa(\texttt{S}) \,|\, \kappa(\texttt{R})) = |\mathcal{M}| \cdot p_r \cdot H_{\text{bin}}(p_s/p_r) = |\mathcal{M}|\, \Lambda(p_s,\, p_r - p_s).$$

1.1.2. The case $p_q > p_s$ (targeted query). When $p_q > p_s$, Alice need not transmit $\kappa(\texttt{S})$ itself—she need only convey enough information for Bob to reconstruct *some* set that contains $\kappa(\texttt{S})$ and is contained in $\kappa(\texttt{Q})$. This additional freedom (the "don't care" region $\kappa(\texttt{Q}) \setminus \kappa(\texttt{S})$) should intuitively make communication cheaper. To prove a lower bound that accounts for this, we use a *rate-distortion* argument.

For any given model $\mu \in \mathcal{M}$, define $X_\mu \in \{0, 1, 2\}$ this way: $X_\mu = 1$ if $\mu \in \kappa(\texttt{S})$ (must be included), $X_\mu = 0$ if $\mu \notin \kappa(\texttt{Q})$ (must be excluded), and $X_\mu = 2$ otherwise ("don't care"). The logical requirement that Bob's reconstruction must contain $\kappa(\texttt{S})$ and be contained in $\kappa(\texttt{Q})$ translates to a per-component *zero-distortion* constraint under a metric ρ that penalizes only violations of the "must include" and "must exclude" conditions.

Choose some set of models $k \subseteq \mathcal{M}$. It turns out that the collection of random variables $\{X_m\}_{m \in k}$ is i.i.d. when conditioned by $\kappa(\texttt{R}) = k$, with distribution $\{1 - p_q/p_r,\ p_s/p_r,\ p_q/p_r - p_s/p_r\}$. Applying the data processing inequality followed by Jensen's inequality (exploiting convexity of mutual information for fixed input and varying channel distributions), one obtains

$$E[\text{cost}] \;\geq\; |\mathcal{M}| \sum_{k \subseteq \mathcal{M}} P(\kappa(\texttt{R}) = k)\, |k| \min_{P_{X,Z} \colon E[\rho(X,Z)]=0} I(X; Z) \;=\; |\mathcal{M}|\, p_r\, \Lambda(p_s/p_r,\, 1 - p_q/p_r).$$

The rate-distortion minimum evaluates to $\Lambda(p_s/p_r, 1 - p_q/p_r)$ via standard variational methods. Averaging over k introduces the factor $E[|k|]/|\mathcal{M}| = p_r$, and the scaling property of Λ provides the final result: $|\mathcal{M}|\, \Lambda(p_s, p_r - p_q)$.

1.2. Upper bound: $\Lambda(p_s, p_r - p_q) + O(|\mathcal{M}|^{-1} \log |\mathcal{M}|)$**.**

1.2.1. The case $p_q = p_s$. Since both parties know $\kappa(\texttt{R})$, Alice need only specify $\kappa(\texttt{S})$ as a subset of $\kappa(\texttt{R})$. She first sends $|\kappa(\texttt{S})|$ using a universal integer code (Elias δ-coding with cost $O(\log |\mathcal{M}|)$ bits), and then sends the index of $\kappa(\texttt{S})$ among all size-$|\kappa(\texttt{S})|$ subsets of $\kappa(\texttt{R})$, requiring at most $\log_2 \binom{|\kappa(\texttt{R})|}{|\kappa(\texttt{S})|} + 1 = \Lambda(|\kappa(\texttt{S})|, |\kappa(\texttt{R})| - |\kappa(\texttt{S})|) + O(1)$ bits. Taking expectations, using the concavity of Λ, and normalizing by $|\mathcal{M}|$ yields the bound.

1.2.2. The case $p_q > p_s$ (partition compression via random coding). The problem becomes one of *partition compression*: Alice must efficiently communicate a partition of the models in $\kappa(\texttt{R})$ that separates the "must include" set $\kappa(\texttt{S})$ from the "must exclude" set $\kappa(\texttt{R}) \setminus \kappa(\texttt{Q})$.

The achievability proof uses a random coding argument inspired by Shannon's original method. Both parties share a random binary matrix C (with infinitely many rows and $|\kappa(\texttt{R})|$ columns), whose entries are i.i.d. with $P(C_{i,j} = 1) = p_s/(p_s + p_r - p_q)$. The point of the matrix C is that it works as a "codebook". For any given row i of C, let k_i denote the set of models obtained through this convention: a 1 indicates that the corresponding element of $\kappa(\texttt{R})$ is part of k_i, and a 0 indicates that it is not.

Alice scans the rows of C from top to bottom until she finds the first row J that meets this condition: $\kappa(\texttt{S}) \subseteq k_J \subseteq \kappa(\texttt{Q})$). The index J is geometrically distributed with a parameter that yields $E[\log_2 J] = |\kappa(\texttt{R})| \cdot \Lambda(p_s/p_r, (p_r - p_q)/p_r)$. Alice sends J using Elias δ-coding; Bob recovers the row and reads off the partition. The expected normalized cost, after taking expectations over the random kernels and applying the concavity of Λ, evaluates to $\Lambda(p_s, p_r - p_q) + O(|\mathcal{M}|^{-1} \log |\mathcal{M}|)$.

Since this is the *expected* performance of a random code, there exists a deterministic code achieving the same bound.

1.3. An example of a deterministic, optimal algorithm for a small problem with targeted queries. In the *Algorithms for targeted queries based on linear algebra* subsection of the main paper, the algorithm presented relied on shared randomness between sender and receiver, and was shown to be optimal in a specific setting using asymptotic arguments. In this subsection, we provide a small example of a deterministic algorithm that is exactly optimal in a slightly different but related sense. The value of this example lies in its simplicity, as it contains most of the essential insights on the problem of targeted queries. We found that, to accomplish this, the GF(2) linear algebra approach of the preceding subsection does not suffice, and a nonlinear construction is required.

We can illustrate this problem with the following scenario. NASA is preparing a mission to Mars. While on Mars, there are several known problems that can arise based on a variety of variables. To keep the list of actions the astronauts must know as simple as possible, the set of remediation actions has been reduced to just 6. There is a catch to this parsimony, however: for each of the known problems, exactly one of the actions will solve the problem, four will have no effect, and one of the actions will cause catastrophic failure. To solve the problem, any number of actions may be taken, as long as a) they are taken simultaneously, and b) they include the correct one and do not include the catastrophic one. Diagnosis of the problem from analyzing the mission's sensor data is performed back in Houston. In response to a diagnosis made in Houston, the space-based transmission back to the astronauts of what action to take must be as efficient as possible – meaning it must take as few bits as possible. Before leaving on the mission, NASA can share whatever information it wishes with the crew in an effort to accomplish

this and agree to a protocol to apply remediation actions. The problem then concerns, what is the minimum number of bits needed to solve this problem? As we will show, this number is 2 bits.

To map the problem to our paper's notation, we identify the correct action by $\kappa(\mathtt{S})$ and the catastrophic action by $\kappa(\mathtt{Q})^c$; therefore we have $|\mathcal{M}| = 6$, $|\kappa(\mathtt{S})| = 1, |\kappa(\mathtt{Q})| = 5$ and where there is no $\mathtt{R}$; as elsewhere in the paper, we have $\kappa(\mathtt{S}) \subseteq \kappa(\mathtt{Q})$. Also as before, the job of the sender is to transmit the minimum number of bits so that the receiver can compute a subset of $\mathcal{M}$ that includes $\kappa(\mathtt{S})$ and is included in $\kappa(\mathtt{Q})$.

We will be using the same definition for X_i as in the previous section. Therefore the vector $X_0X_1X_2X_3X_4X_5$ has exactly one 0, one 1, and four 2s. One way to send a partition that separates the (single entry) sets $\{i : X_i = 0\}$ and $\{i : X_i = 1\}$ is to send an integer in the set $\{0, 1, 2, 3, 4, 5\}$ identifying the one element in, say, the first set. The cost of this is $\log_2 6$ bits. Alternatively, one can scan each of the 4 vectors below to find one that matches with $X_0X_1X_2X_3X_4X_5$ on the 0s and 1s, regarding the 2s as "don't care":

	X_0	X_1	X_2	X_3	X_4	X_5
first vector	0	0	0	1	1	1
second vector	0	1	1	0	1	0
third vector	1	0	1	1	0	0
fourth vector	1	1	0	0	0	1

We observe that any two columns of this binary matrix contain at least one row with the pattern "0 1" and one row with the pattern "1 0". For example, if $X_0 = 1, X_1 = 2, X_2 = 2, X_3 = 2, X_4 = 0, X_5 = 2$, then the third and fourth vectors are valid vectors. Therefore, one such vector can always be found, and it can be specified using $2 < \log_2 6$ bits.

It is possible to obtain, for any given desired length $|\mathcal{M}|$, a crude bound on the minimum number of rows t in a $t \times |\mathcal{M}|$ binary matrix that partitions two sets each comprising exactly one non-overlapping integer in $\{0, \ldots, |\mathcal{M}| - 1\}$. Take any one row of this matrix, and assume it has n_0 zeros and n_1 ones. The number of patterns with exactly one 0, one 1, and the rest don't cares, that can be handled by any one row is at most $n_0 n_1 \leq |\mathcal{M}|^2/4$. Therefore the entire matrix with t rows can handle at most $t|\mathcal{M}|^2/4$ such patterns. There are a total of $|\mathcal{M}|(|\mathcal{M}| - 1)$ patterns that we need to handle, and therefore the following relation must always hold:

$$t \geq 4\left(1 - \frac{1}{|\mathcal{M}|}\right).$$

Rounding up (since t is an integer), we see that at least 4 rows are needed for any value of $|\mathcal{M}| \geq 5$, showing that the deterministic algorithm for the above small example is optimal in this sense.

2. Case 2: Alice does not know $\mathtt{R}$

When Alice does not know what Bob knows, the communication problem is fundamentally different: Alice cannot tailor her message to Bob's background. To guarantee that Bob will be able to prove what Alice can prove, Alice and Bob must *interact*—exchanging messages over multiple turns—so that Bob's responses can implicitly reveal enough about his background to make Alice's transmissions efficient. In this setting we restrict to $\mathtt{Q} = \mathtt{S}$ (i.e., $p_q = p_s$), and the figure of merit accounts for all bits exchanged in both directions.

This interactive setting is inspired by the Slepian–Wolf theorem in classical information theory, which shows that a receiver's side information can reduce the required communication rate even when the sender does not know that side information.

2.1. Lower bound: $\Lambda(p_s, p_r - p_s)$. This setting can be directly compared to that of Case 1 when $p_q = p_s$. Intuitively the absence of $\mathtt{R}$ on Alice's side can only make things harder compared to the Case 1 we treated, and therefore, whatever cost we must pay must be at least that of Case 1. The reader may notice that the class of protocols in Case 2 is strictly more powerful: we allow interaction, whereas in Case 1 we did not. Alas, the detailed mathematical argument (1), shows that this additional power does not buy us anything.

2.2. Upper bound: $\Lambda(p_s, p_r - p_s) + O(|\mathcal{M}|^{-1} \log |\mathcal{M}|)$. We construct an explicit four-turn protocol, inspired by Slepian–Wolf hashing, that achieves the optimal rate as follows.

1. *Bob → Alice* (Turn 0): Bob sends $|\kappa(\mathtt{R})|$ using Elias δ-coding. Cost: $O(\log |\mathcal{M}|)$ bits.

2. *Alice → Bob* (Turn 1): Having received $|\kappa(\mathtt{R})|$, both parties can now independently compute a target rate:

$$\text{RATE} = \Lambda\left(\frac{|\kappa(\mathtt{S})|}{|\mathcal{M}|}, \frac{|\kappa(\mathtt{R})| - |\kappa(\mathtt{S})|}{|\mathcal{M}|}\right) + \frac{\log_2 |\mathcal{M}|}{|\mathcal{M}|}.$$

   Alice sends $|\kappa(\mathtt{S})|$ (Elias coded) together with the *hash bin index* of $\kappa(\mathtt{S})$ under a pre-agreed random hash function at this rate. Cost: $|\mathcal{M}| \cdot \text{RATE}$ bits (dominant term).

3. *Bob decodes* (Turn 2): Bob searches his bin for a unique kernel k of the correct size $|\mathtt{S}|$ that satisfies $k \subseteq \kappa(\mathtt{R})$—the logical constraint that any valid sender kernel must be contained in the receiver's kernel. If Bob finds one such thing, he succeeded, if not, he failed. He signals success or failure with 1 bit.

4. *Fallback* (Turn 3): If decoding failed, Alice sends $\kappa(\mathtt{S})$ directly ($|\mathcal{M}|$ bits).

Why does this work? The key insight is that Bob possesses powerful *side information*: he knows $\kappa(\texttt{R})$ and can therefore reject any hypothesis k in the hash bin that is not a subset of $\kappa(\texttt{R})$. This dramatically reduces the effective number of competing hypotheses. The role of Step 2 is to do a more delicate tuning of how much information Alice has to send to Bob given the sizes of the kernels that both Alice and Bob have.

Error probability analysis. A decoding failure occurs when a "confounding" kernel, namely one that is the right size $|\kappa(\texttt{S})|$, lands in the same hash bin as $\kappa(\texttt{S})$. By independence of the random bin assignments, we have

$$P(\text{failure}) \leq 1 - \left(1 - 2^{-|\mathcal{M}|\,\text{RATE}}\right)^{\binom{|\kappa(\texttt{R})|}{|\kappa(\texttt{S})|}}.$$

The design of RATE ensures that $\binom{|\kappa(\texttt{R})|}{|\kappa(\texttt{S})|} \leq 2^{|\mathcal{M}|\,\text{RATE} - \log_2|\mathcal{M}|}$ (since $\log_2\binom{r}{s} \leq r\,H_{\text{bin}}(s/r) = \Lambda(s, r-s)$). Applying Bernoulli's inequality $(1-a)^n \geq 1 - an$, we obtain

$$P(\text{failure}) \leq 2^{-|\mathcal{M}|\,\text{RATE}} \cdot \binom{|\kappa(\texttt{R})|}{|\kappa(\texttt{S})|} \leq \frac{1}{|\mathcal{M}|}.$$

The total expected normalized cost is therefore given by

$$\text{RATE} + \frac{1}{|\mathcal{M}|} + \frac{P(\text{failure}) \cdot |\mathcal{M}|}{|\mathcal{M}|} + O\left(\frac{\log|\mathcal{M}|}{|\mathcal{M}|}\right) = \Lambda(p_s, p_r - p_s) + O\left(\frac{\log|\mathcal{M}|}{|\mathcal{M}|}\right),$$

where the $P(\text{failure}) \cdot |\mathcal{M}|/|\mathcal{M}| \leq 1/|\mathcal{M}|$ term accounts for the fallback. As before, since this is the expected performance of a random hash function, a deterministic hash function achieving the same bound must exist.

3. Unification and discussion

Both cases of the theorem are unified by the single expression $\Lambda(p_s, p_r - p_q)$:

- When Alice knows R and can target a specific query (Case 1), the cost is $\Lambda(p_s, p_r - p_q)$ for any $p_q \in [p_s, p_r)$.
- When Alice does not know R and communicates everything she can (Case 2, $p_q = p_s$), setting $p_q = p_s$ in the same expression recovers $\Lambda(p_s, p_r - p_s)$.

It is noteworthy that the *interactive* protocol of Case 2 achieves the same asymptotic rate as the *one-shot* protocol of Case 1 (when $p_q = p_s$). This shows that Alice's ignorance of Bob's background knowledge R does not fundamentally increase the communication cost—the interaction compensates exactly for the missing knowledge, in the spirit of the Slepian–Wolf theorem, but in a setting that is lossy (Bob does not have to reconstruct the same sentence Alice has, just a suitable sentence).

3.1. The advantage of reasoning over naïve transmission. The limit $\Lambda(p_s, p_r - p_q)$ should be contrasted with what a system *without* reasoning capabilities would require. For simplicity, assume that the receiver has prior information (and hence $p_r = 1$). A naïve approach that treats $\kappa(\texttt{S})$ as a classical information source requires $H_{\text{bin}}(p_s)$ bits after normalization by $|\mathcal{M}|$ (the Shannon entropy of the kernel). The gap

$$H_{\text{bin}}(p_s) - \Lambda(p_s, 1 - p_q)$$

quantifies the communication savings made possible by the receiver's ability to reason with background knowledge R and the entailment structure $\texttt{S} \vdash \texttt{Q} \vdash \texttt{R}$. This gap, which one may construe as a semantic advantage, is strictly positive whenever $p_q > p_s$ (targeted queries), vanishing only when $p_q = p_s$.

3.2. Role of the i.i.d. assumption in the lower bounds. In the *Conditions for the validity of the lower bound of Theorem 1* in the Experimental section of the main paper, we introduced an i.i.d. assumption for our lower bounds to hold. There are many equivalent ways to describe such assumption; in one perspective, it says that whenever a kernel is assumed to be included in another kernel, the models in the former are chosen i.i.d. from the models in the latter. This assumption enters the proof at two critical junctures, both in Section 2.1.1:

1. *Decomposition into per-component entropies*: For the setting where R is known to both Alice and Bob, and in the case $p_q = p_s$, the i.i.d. assumption allows the conditional entropy $H(\kappa(\texttt{S})|\kappa(\texttt{R}))$ to be written as a sum of binary entropies—without independence, the entropy could be smaller due to correlations.
2. *Application of Jensen's inequality in the rate-distortion argument*: For the setting where $p_q > p_s$, the i.i.d. assumption allows Jensen's inequality to extract a single rate-distortion problem from the sum of per-component mutual informations.

Without the i.i.d. condition, structured correlations between kernel elements could in principle allow more efficient coding, and the lower bound $\Lambda(p_s, p_r - p_q)$ need not hold. However, the upper bounds are free of this restriction: they hold for *any* distribution over $(\texttt{S}, \texttt{Q}, \texttt{R})$ satisfying the entailment conditions and with (p_s, p_q, p_r) expected kernel sizes. Thus the theorem establishes $\Lambda(p_s, p_r - p_q)$ as both the best achievable rate in generality and the fundamental limit under the i.i.d. model. Complete details are provided in (1).

4. Additional details on experimental methodology

4.1. Method of Generating Kernels. Since the sender's kernel is a subset of the receiver's kernel, and, moreover, the query is provable by the sender but not the receiver, it follows that the sender's kernel is contained in the kernel associated with the query, which in turn is contained in the receiver's kernel. It then follows that we can go through all the possible truth-value assignments for the 10 variables and, for each assignment μ, generate a random number $\eta \in [0, 1]$ where, if $\eta \leq p_r$, we place μ in the receiver's kernel. If additionally $\eta \leq p_q$, then we can place μ in the query kernel, and if additionally to that $\eta \leq p_s$, then we place μ in the sender's kernel.

4.2. Method of Generating Generalized Decision Tree Sentences. The gist of the decision tree approach is to recursively call a method $\texttt{GENERATE\_GDT\_FOR\_KERNEL}(\cdot)$ to find a most balanced variable $\texttt{Z}_\texttt{b}$ among the variables $\texttt{Z}_1, ..., \texttt{Z}_\texttt{n}$ – meaning a variable where $\texttt{Z}_\texttt{b} = 1$ and $\texttt{Z}_\texttt{b} = 0$ as close to equally as possible among the kernel elements – and then output

$$(\texttt{Z}_\texttt{b} \wedge \texttt{GENERATE\_GDT\_FOR\_KERNEL}(\texttt{pos\_kernel})) \vee$$
$$(\neg\texttt{Z}_\texttt{b} \wedge \texttt{GENERATE\_GDT\_FOR\_KERNEL}(\texttt{neg\_kernel})),$$

where $\texttt{pos\_kernel}$ is the reduced kernel on the $\texttt{n} - \texttt{1}$ variables $\texttt{Z}_1, ..., \texttt{Z}_\texttt{n}$ but with $\texttt{Z}_\texttt{b}$ excluded, and in the original kernel $\texttt{Z}_\texttt{b} = 1$, while $\texttt{neg\_kernel}$ is the reduced kernel on the $\texttt{n} - \texttt{1}$ variables $\texttt{Z}_1, ..., \texttt{Z}_\texttt{n}$ but with $\texttt{Z}_\texttt{b}$ excluded, and in the original kernel $\texttt{Z}_\texttt{b} = 0$. The generalized version of this heuristic is a bit more nuanced: variables that are effectively constant with respect to the kernel (in other words, there is a variable $\texttt{Z}_\texttt{i}$ such that either $\texttt{Z}_\texttt{i} = 1$ or $\texttt{Z}_\texttt{i} = 0$ for every kernel element) are efficiently split out; and when there are $\texttt{k}$ variables remaining and the kernel size is either $2^\texttt{k}$ or 0, the routine immediately terminates, outputting an empty string (equivalent to outputting True) in the former case and outputting a False indicator in the latter case. There is a considerable literature on the use of decision trees to represent Boolean functions. See, for example, (2–4).

References

1. LA Lastras, et al., Towards a unification of logic and information theory (2024) https://arxiv.org/abs/2301.10414.
2. Y Breitbart, H Hunt, D Rosenkrantz, On the size of binary decision diagrams representing boolean functions. *Theor. Comput. Sci.* **145**, 45–69 (1995).
3. D Mehta, V Raghavan, Decision tree approximations of boolean functions. *Theor. Comput. Sci.* **270**, 609–623 (2002).
4. R O'Donnell, M Saks, O Schramm, R Servedio, Every decision tree has an influential variable in *46th Annual IEEE Symposium on Foundations of Computer Science (FOCS'05)*. pp. 31–39 (2005).
